# Modelling of the frictional behaviour of the snake skin covered by anisotropic surface nanostructures

**DOI:** 10.1038/srep23539

**Published:** 2016-03-23

**Authors:** Alexander E. Filippov, Stanislav N. Gorb

**Affiliations:** 1 Department of Functional Morphology and Biomechanics, Kiel University, Am Botanischen Garten 1–9, D-24118 Kiel, Germany; 2 Donetsk Institute for Physics and Engineering, National Academy of Sciences of Ukraine, Donetsk, Ukraine

## Abstract

Previous experimental data clearly revealed anisotropic friction on the ventral scale surface of snakes. However, it is known that frictional properties of the ventral surface of the snake skin range in a very broad range and the degree of anisotropy ranges as well to a quite strong extent. This might be due to the variety of species studied, diversity of approaches used for the friction characterization, and/or due to the variety of substrates used as a counterpart in the experiments. In order to understand the interactions between the nanostructure arrays of the ventral surface of the snake skin, this study was undertaken, which is aimed at numerical modeling of frictional properties of the structurally anisotropic surfaces in contact with various size of asperities. The model shows that frictional anisotropy appears on the snake skin only on the substrates with a characteristic range of roughness, which is less or comparable with dimensions of the skin microstructure. In other words, scale of the skin relief should reflect an adaptation to the particular range of surfaces asperities of the substrate.

The locomotion without extremities has important tribological consequences in snakes, because their ventral body surface is almost in continuous contact with the substrate. In fact, in order to facilitate locomotion, the surface of the snake skin has to generate low friction, supporting sliding in the forward direction, and simultaneously produce high friction, enabling propulsive force generation along the substrate^1^,^2^. Generally, frictional properties of two materials in contact depend on various factors, such as surface energy and material properties of both surfaces, but one of the most important parameters is the surface roughness of both bodies in contact^3^,^4^. Thus, in the case of the snake, the specific roughness of the substrate, on which the snake moves, must play a very important role in generation of frictional forces. Additionally, the ventral surface of the snake is also not smooth, but consists of caudally-oriented scales (Fig. 1A,C), which are covered with very specific caudally-oriented kind of nanostructure, so called microdermatoglifics (Fig. 1B,D,E,F)^1^,^2^,^5^,^6^,^7^,^8^,^9^,^10^,^11^,^12^,^13^,^14^,^15^,^16^, and the previous authors suggested that the specific ventral surface of the snake skin is of high relevance for the anisotropic friction generation and thus facilitation of snake locomotion. Previous atomic force microscopy and confocal laser scanning microscopy studies revealed non symmetric, but regular denticle-like nanostructures on ventral scales of the vast majority of snake species. The structures are 2.46 ± 0.45 μm long, 0.60 ± 0.11 μm wide) and oriented in caudal direction and parallel to the longitudinal body axis^1^ (Fig. 1B,D,E,F).

In the Fig. 1E,F, we demonstrate ventral skin surface nanostructures of two species of snakes belonging to two different families. The black-necked spitting cobra *Naja nigricollis* (Elapidae) inhabits savannas and semi-desert regions of Africa. They also live in coastal scrubs and dry grasslands. They usually move on the ground but are also excellent tree climbers. The western diamondback rattlesnake *Crotalus atrox* (Viperidae) inhabits wide range of habitats from flat plains to rocky canyons in the United States and Mexico. It lives on the ground in the sandy areas, grassland, scrub, pine-oak forests, however, they are poor climbers. The detailed data on the morphology of the skin surface nanostructure in numerous other species of snakes is provided in earlier publications^1^,^6^.

Previous experimental data using various tribological approaches clearly revealed anisotropic friction on the ventral scale surface of snakes^1^,^14^,^15^,^16^,^17^,^18^. Meanwhile there are biomimetic surface structures with anisotropic friction^19^,^20^,^21^ inspired by the micro-20 and nanostructures^19^,^21^ of the snake skin. However, it is known that frictional properties of the ventral surface of the snake skin range in a very broad range and the degree of anisotropy ranges as well to a quite strong extent. This might be due to the variety of species studied, diversity of approaches used for the friction characterization (AFM, microtribometer, sliding test on the slope), and/or due to the variety of substrates used as a counterpart in the experiments. In order to understand, and may be even predict the interactions between the nanostructure arrays of the ventral surface of the snake skin, this study was undertaken, which is aimed at numerical modeling of frictional properties of the structurally anisotropic surfaces in contact with various size of asperities. Previously, in the numerical experiment, we showed the effect of stiffness of the surface structures on frictional anisotropy^22^, whereas in the present work we concentrated on the role of relative dimensions between skin structures and substrate roughness in friction generated in different sliding directions.

## Numerical modeling

To simulate anisotropy of friction of the skin covered with anisotropic microstructures, we used an appropriate modification of Tomlinson-Prandtl (TP) model. According to the above observations, the skin is covered by slightly randomized periodic structure of the asymmetric holes with short relatively deep slopes from one side and long smooth slopes from another. One of the simplest ways to mimic such a structure in numerical simulation is to use an array of almost periodically placed Gaussians with slightly randomized (negative) amplitude and positions, having different widths in two opposite directions:


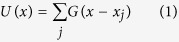


Here index *j* = 1, 2, …, *N* numerates the positions of the minimums. It runs along all the system. Total length of the system is defined by the condition 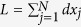. The distances between minimums of nearest Gaussians are determined by the array: *dx*_j_ = *dx*_0_(1+*ζ*_*j*_). The statement that system is almost periodic means that the distance between minimums is varied around average value *dx*_0_ = *const* = *L/N* by *δ*-correlated random deviations *ζ*_*j*_, and these variations are relatively small:


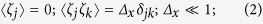


The anisotropic Gaussians are defined by the following formulae:


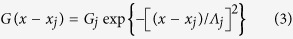


where *G*_*j*_ and Λ_*j*_ are randomized in the same manner as *dx*_*j*_ with corresponding parameters Δ_G,Λ_<<1. Besides, to reproduce realistically observable anisotropic forms of the surface Eq.1 we take different widths in positive and negative directions:


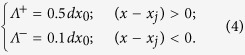


**Figure 2:**
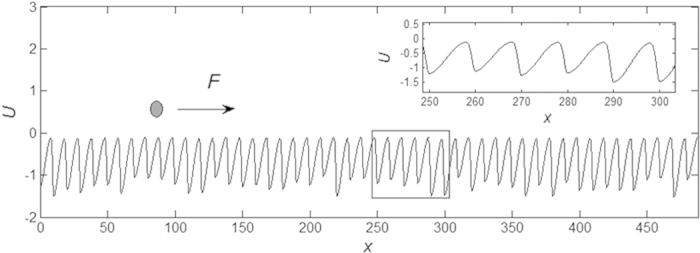
Conceptual structure of the model. The potential *U*(*x*) = ∑_*j*_*G*(*x* − *x*_*j*_) is constructed using an array of anisotropic Gaussians. The probe (which represents an asperity of the substrate) driven by external force is shown by a dark circle. It can be either zero-size point, or a body of different size, up to the sizes comparable with the period of the potential. The anisotropy of the Gaussians is shown for a small fragment of the surface (marked by the rectangle) and enlarged in the inset.

Typical form of the randomized effective potential *U*(*x*) obtained after accumulation of the Gaussians in Eq.1 with account of all the conditions of Eq. 2, is shown in the concept image (Fig. 2). If the probe, used in standard TP friction model, is a zero-size point (shown by black dot in Fig. 2), this potential causes effective force *f*_surf_ (*X*)=−∂*U*(*x*)/∂*x|*_*x=X*_, which acts in the equations of motion:


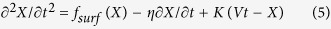


where *V*, *K* and *η* are velocity, elastic and damping constants of an external spring, driving the probe with an instant coordinate *X*, respectively.

More realistic variant of the model corresponds to a limited (non-zero) size *θ* of the body, which simulates a characteristic size of the asperities interacting with surface potential *U*(*x*). In this case total surface force *f*_*surf*_ (*X*) is equal to an integral of the all impacts accumulated along all the segments of the surface taken in interval |*X*−*x*| < *θ*:


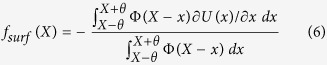


with a kernel, which monotonously decreases with distance from the center of the probe body |*X*−*x*|. For the characteristic size *θ* it is quite self-consistent to take simply 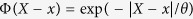.

One can expect that in the limit *θ* << *dx*_0_ the model will reduce to the case of zero-size body, where anisotropy of the surface is most pronounced. For opposite inequality *θ* > *dx*_0_ the body will cover some number of the periods of the system. As result, the anisotropy will become less pronounced and in strong limit *θ* >> *dx*_0_ the body will tread the surface as practically flat and symmetric in both directions.

## Results and Discussion

**Figure 3:**
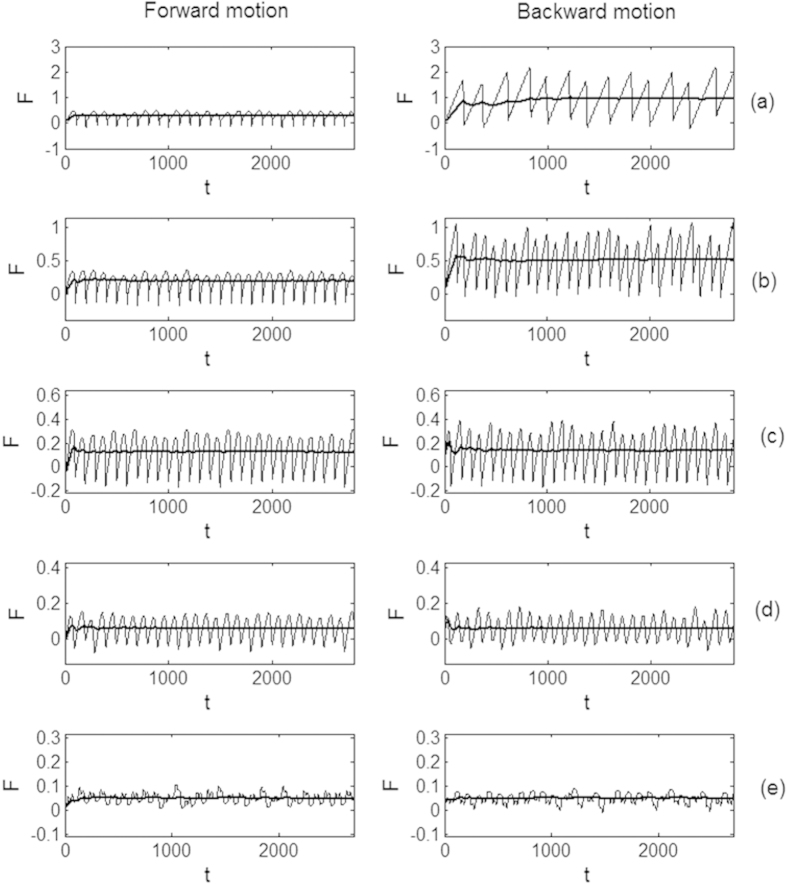
Typical time-depending friction forces for forward and backward directions of motion, shown in left and right panels of the subplots (**a–e**) for 5 representative sizes of the probe *θ* = {.01Λ^−^, Λ^−^, (Λ^−^ + Λ^+^)/2, Λ^+^, 3Λ^+^/2}, respectively. Bold lines in all the plots correspond to the time averaged friction forces. Please, notice different vertical axes, which monotonously decrease from (**a**) to (**e**) subplots (but coincide for every pair of left and right plots). See supplementary video 1.

Our simulations generally confirm these expectations. The results are summarized in Fig. 3. We present friction force for 5 representative values:


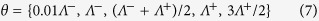


of the width *θ* in subplots (a–e), respectively. Left and right panels for each of the cases (a–e) reproduce the force for positive and negative directions of motion, respectively. Well pronounced qualitative difference between these two directions for the first 3 cases is seen directly: strong stick-slip behavior in one direction and smooth motion in another. Besides, we calculate instant time-averaged mean friction.


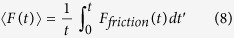


**Figure 4:**
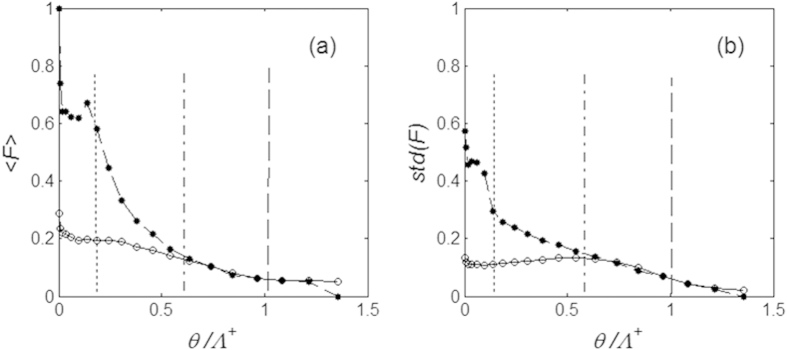
Dependence of the mean friction forces (**a**) and standard deviations (**b**) for forward and backward directions of motion, marked by white and black circles, respectively, calculated in the interval of probe sizes corresponding to the representative values, shown in Fig. 3. Dotted, dash-dotted and dashed lines mark the cases: *θ* = Λ^−^, *θ* = (Λ^+^ + Λ^−^)/2 and *θ* = Λ^+^, respectively. See supplementary video 2.

It is shown in all the cases by the bold lines (Fig. 4). For the stationary process here, when time is going to infinity *t* → ∞, it tends to a constant value <*F*(*t*) > → <*F>* = *const*., which is depending on the size of the probe/asperity. At relatively small probes <*F* > for positive direction of the motion is considerably higher than in the opposite direction.

It is interesting to note that already relatively medium size of the probe, which in reality will correspond to the surface asperity, from *θ* = Λ^+^ to *θ* = 1.5Λ^+^ (having an order of period *dx*_0_) leads to a practically complete smothering of the friction curves in both directions, which was preliminary expected for the larger *θ* >> *dx*_0_. It means that, frictional anisotropy appears on the snake skin ventral surface only on the substrates with a characteristic range of roughness, which is less or comparable with dimensions of the skin microstructure. In other words, scale relief should reflect an adaptation to the particular range of surfaces asperities of the substrate. However, in the case, when many substrate asperities interact simultaneously with the skin surface, the stick-slip behavior might be not that strongly pronounced as in the present model. It can be even lower due to the random distribution of denticle tips on the snake scale^1^.

In the literature, there are only several studies that report on frictional properties of snake skin on different roughness^15^,^16^,^17^,^18^. Berthé *et al.*^15^ performed frictional experiments on different scales of *Corallus hortulanus*, in three directions on nine different rough surfaces and showed frictional anisotropy along the rostro-caudal body axis and along the medio-lateral one on all tested substrate roughnesses. In other study, rough spheres with *R*_*a*_ = 4 μm^16^ and 2.4 μm^1^ were applied as a sliding probe. Friction coefficient obtained in the cranial direction was always significantly lower than that in the caudal direction. An estimation of the frictional behavior of snake skin on rigid styrofoam material^18^ also showed anisotropic frictional properties in forward and backward directions.

Some enhancement of frictional anisotropy was also found in our previous experiments for cushioned (soft underlying layer) skin of the snake *Lampropeltis getula californiae* versus uncushioned one (rigid underlying layer) in contact with rough rigid substrate. The comparison of frictional experiments with anesthetized snakes on relatively smooth and rough surfaces (*R*_*a*_ = 20 and 200 μm, respectively) demonstrated frictional anisotropy, which almost completely disappeared on the smooth surface^17^. However, these latter experiments presumably show the effect of interlocking of individual scales on such a coarse roughness. Thus, based on the previous literature data and results of our numerical modeling, presented here, we can assume that particular dimension of the nanostructure on the ventral scales adapted to enhance frictional anisotropy at nanoscale substrate roughness. The frictional anisotropy at the macro- and microscale is provided by the macroscopic pattern of ventral scales. One can conclude that the frictional anisotropy of the ventral surface is provided by two hierarchical levels of structures: scales and denticles.

This is the reason why snakes strongly decrease their locomotory ability on smooth substrates and always rely on certain dimension of roughness (and even nanoscale roughness, where scales cannot be used, might be sufficient for generating propulsion), the fact which is perfectly in agreement with the numerical model presented in this study.

## Additional Information

**How to cite this article**: Filippov, A. E. and Gorb, S. N. Modelling of the frictional behaviour of the snake skin covered by anisotropic surface nanostructures. *Sci. Rep.* **6**, 23539; doi: 10.1038/srep23539 (2016).

## Supplementary Material

Supplementary Information

Supplementary Video 1

Supplementary Video 2

## Figures and Tables

**Figure 1:**
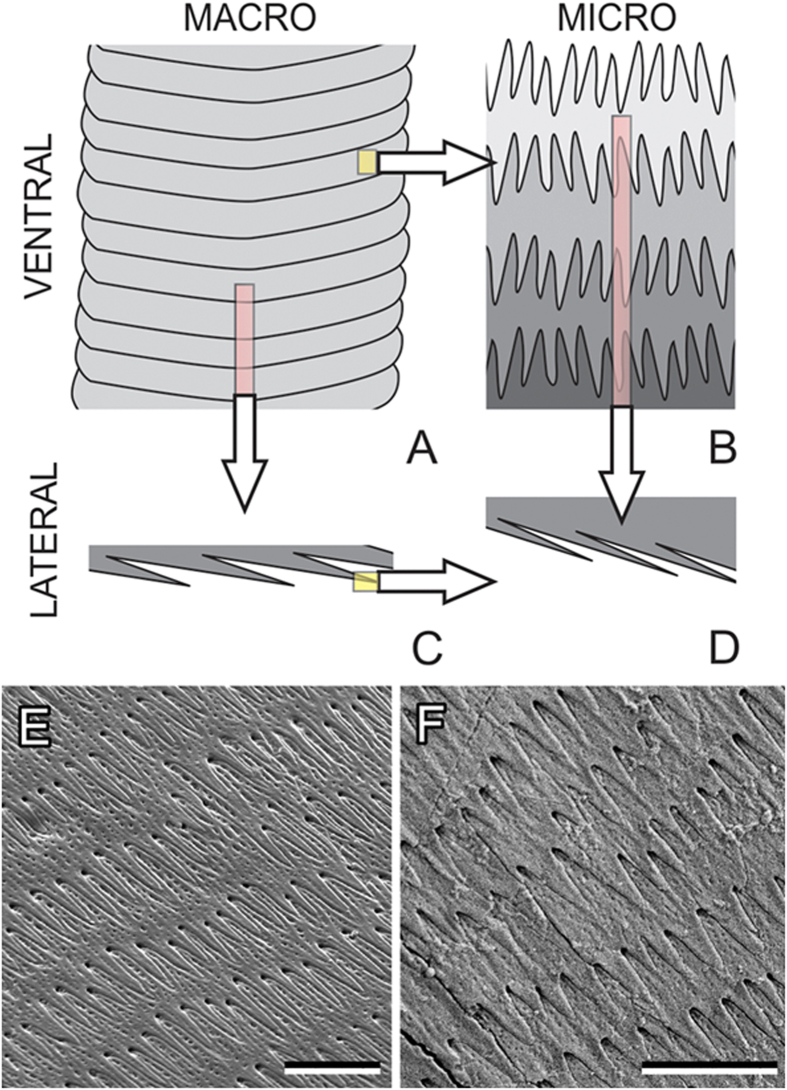
(**A–D**) Hierarchical anisotropic structures on the ventral snake surface (upper corner of the images points towards head). (**A,C**) Ventral scales (macroscopic dimension). (**B,D**) Denticles or micrornamentation at the scale surface (microscopic to nanoscopic dimensions). (**A,B**) Ventral aspect. (**C,D**) Lateral aspect (side view). (**E,F**) Scanning electron micrographs of the ventral snake skin. (**E**) Western diamondback rattlesnake *Crotalus atrox* (scale bar: 5 μm). (**F**) Black-necked spitting cobra *Naja nigricollis* (scale bar: 5 μm). Left upper corner of images E and F points towards head. Denticles are caudally oriented.
