# Supplementary material for: Modelling of the frictional behaviour of the snake skin covered by anisotropic surface nanostructures
Source: Sci Rep. 2016 Mar 23;6:23539. doi: 10.1038/srep23539 (PMC4804221; doi:10.1038/srep23539)
Supplement: Supplementary Information [file srep23539-s1.pdf]

Modelling of the frictional behaviour of the snake skin covered by anisotropic surface nanostructures

Alexander E. Filippov and Stanislav N. Gorb

Supplementary videos

Video 1. Typical time-depending friction forces for two forward and backward directions of motion, shown in left and right panels of the subplots for 5 representative sizes of the probe $\theta = \{.01\Lambda^{-},\ \Lambda^{-},\ (\Lambda^{-}+\Lambda^{+})/2,\ \Lambda^{+},\ 3\Lambda^{+}/2\}$, respectively. See also Figure 3 of the paper.

Video 2. Dependence of the friction forces for forward and backward directions of motion, calculated in the interval of probe sizes corresponding to the representative values, shown in Fig. 3. See also Figure 4 of the paper.
